# Trends in comorbid physical and mental health conditions in children from 1999 to 2017 in England

**DOI:** 10.1007/s00787-022-02112-5

**Published:** 2022-11-23

**Authors:** Laura Panagi, Tamsin Newlove-Delgado, Simon R. White, Sophie Bennett, Isobel Heyman, Roz Shafran, Tamsin Ford

**Affiliations:** 1 Department of Psychiatry, University of Cambridge, The Clifford Allbutt Building, Biomedical Innovation Hub/Bay 13, Cambridge Biomedical Campus, Hills Road, Cambridge, CB2 OAH UK; 2 College of Medicine and Health, University of Exeter, St Luke’s Campus, Heavitree Road, Exeter, EX1 2LU UK; 3 UCL Great Ormond Street Institute of Child Health, 30 Guilford Street, London, WC1N 1EH UK

**Keywords:** Trends, Long-term physical health conditions, Mental health conditions, Comorbitidy

## Abstract

**Introduction:**

Children with long-term physical health conditions (pLTCs) are at increased risk of mental health conditions but less is known about time trends in the mental health of this group of children.

**Methods:**

We used data from three comparable, population-based surveys of children conducted in 1999, 2004, and 2017. We examined whether the proportion of children aged 5–15 years old with comorbid mental health conditions (measured using the multi-informant Development and Well-being Assessment tool) and pLTCs (measured using parental report) in England increased from 1999 to 2017 using linear regression analysis.

**Results:**

Our analysis used data from 8662 (1999), 6401 (2004) and 6219 (2017) children, respectively. The proportion of children with comorbid pLTCs and psychiatric disorders was 0.050 (95% CI = 0.045, 0.055) in 1999, 0.054 (95% CI = 0.049, 0.060) in 2004, and 0.059 (95% CI = 0.053, 0.065) in 2017. The linear regression model revealed a non-significant effect of time on the proportion of children with comorbid pLTCs and psychiatric disorders from 1999 to 2017 (*B* = 0.0004785; SE = 0.0001256; *p* = 0.163).

**Conclusion:**

The estimated prevalence of school-aged children with comorbid pLTCs and mental health conditions in England remained stable since 1999, highlighting the need to prioritize mental health resources for children with physical health comorbidities.

**Supplementary Information:**

The online version contains supplementary material available at 10.1007/s00787-022-02112-5.

## Introduction

Globally, there is an increased focus on the importance of children and young people’s (CYP) mental health [1]; however, less is known about the mental health of CYP with long-term physical health conditions (pLTCs) over time. Research indicates that the risk of having a mental health disorder is two–four times greater in children with pLTCs as compared to their physically healthier counterparts [2]. Untreated mental health conditions in pediatric patients are linked with suboptimal disease management [3], negative health outcomes [4, 5], increased hospitalization stay, and costs compared to peers without a mental health comorbidity [6, 7]. Therefore, there is an urgent need to estimate and respond to the mental health needs of young patients with mental health disturbances.

Studies of clinical samples reflect only those who are in contact with services and whose mental health condition has been detected. An understanding of the level of comorbid pLTCs and poor mental health in all CYP using robust, population-based data is essential for health care provision planning and commissioning, as well as development of health policy. However, no population-based studies have investigated time trends in comorbid pLTCs and mental health conditions in CYP. We aimed to examine whether the proportion of CYP with comorbid mental health conditions and pLTCs in England increased from 1999 to 2017 using population-based, national surveys.

## Methods

### Participants

We used data from the British Child and Adolescent Mental Health Surveys and the Mental Health of Children and Young People in England Survey. These are cross-sectional, population-based surveys of CYP conducted in 1999, 2004, and 2017. The 1999 (age 5–15 years; Great Britain) and 2004 (age 5–16 years; Great Britain) samples were drawn from the Child Benefit Register. The 2017 sample (age 2–19 years; England) was drawn from the NHS Patient Register, as Child Benefit was no longer universal. A detailed description of all three surveys is reported elsewhere [8]. The 1999 and 2004 data are publicly available via the UK Data Service (references 4227 and 5269, respectively), and the 2017 data were accessed via NHS Digital’s Data Access Request Service (DARS, reference DARS-NIC-424336-T7K7T-v0.6). The original surveys were approved by Research Ethics Committees [9, 10], while the University of Cambridge Ethics Committee does not require applications for secondary data analysis. Informed consent was obtained from legal guardians (for children < 11 years old) and from adolescent participants.

In all three surveys, data were collected from parents (94% from the biological mothers), children (aged ≥ 11 years), and teachers (the family nominated the teacher who knew the child best). Parents and CYP were interviewed face-to-face by trained lay interviewers using computer-assisted interviews, with self-reported questionnaires completed for sensitive topics. Teachers responded to a mailed questionnaire. To increase the comparability between the three surveys, we included children from England only (excluded *n* = 2514) aged 5–15 years old (excluded *n* = 3502). Participants with incomplete data on study measures were also excluded (*n* = 234).

### Measures

Mental health conditions were measured using the validated Development and Well-being Assessment [11], a standardized diagnostic tool that combines highly structured and semi-structured questions about psychiatric disorders, based on the International Classification of Diseases-10 (ICD-10) criteria [12]. The DAWBA incorporated information from parents (via interview), CYP (via interview), and teachers (via questionnaire). Computer-generated predictions of disorders were produced and reviewed by trained clinicians (including co-authors TF and TND) who assigned diagnoses based on ICD-10 criteria [12]. The DAWBA covered the following: (emotional disorders [separation anxiety disorder, specific phobia, social phobia, panic disorder, agoraphobia, post-traumatic stress disorder, obsessive compulsive disorder, generalized anxiety disorder, other anxiety disorder, depressive episode, other depressive episode], hyperactivity disorders [hyperkinetic disorder, other hyperactivity disorder], conduct disorders [oppositional defiant disorder, conduct disorder confined to family, unsocialized conduct disorder, socialized conduct disorder, other conduct disorder], less common psychiatric disorders [autistic spectrum disorder, tic disorder, eating disorder, selective mutism, psychosis]).

pLTCs were assessed using parent report on whether the child has any of the following conditions at the time of assessment (yes/no): asthma, eczema, hayfever, epilepsy, cerebral palsy, muscle disease, coordination problems, heart problems, food allergy, kidney/urinary tract problems, a condition present since birth (e.g., club foot or cleft palate), deformities, spina bifida, cystic fibrosis, blood disorders, missing limb(s), diabetes, cancer, vision problems, hearing problems. This selection was based on the consensus definition of chronic health conditions in childhood [13] and discussion with our Study Steering Committee.

### Statistical analysis

We estimated the population prevalence of CYP with comorbid pLTCs and mental health conditions in 1999, 2004, and 2017. We also estimated the population prevalence of children without identified pLTCs or mental health conditions (healthy group), with pLTCs but no mental health conditions, and with mental health conditions but no pLTCs at the three time points. Prevalence analyses used survey weighting (provided by the three national surveys) to account for selection and non-response bias [8]. Not all teachers responded to the mailed questionnaire [14]; For the 2017 survey, weighting does not fully adjust for the teacher report sub-sample. Nevertheless, applying the teacher adjustment factor, provided for the group of children with mental health conditions [14], had minimal impact relative to our estimated 95% Confidence Interval (CI). Given that the teacher adjustment factor is provided for children with mental health conditions and it is not broken down for our sub-groups (children with pLTCs only, children with mental health conditions only, children with comorbid pLTCs and mental health conditions, healthy children), by applying this factor would not give correct estimates.

In the sub-group of children with comorbid pLTCs and mental health conditions, we undertook linear regression analysis to test for a linear trend in survey weighted prevalence across the three surveys (the trend analysis was weighted by the standard errors for each survey to account for uncertainty in the estimates). In sensitivity analyses, we examined whether there were significant changes in the proportion of children with comorbidities by including the whole Great Britain sample (the 1999 and 2004 surveys included children from Scotland and Wales, while the 2017 survey only sampled from England). Analyses were carried out in STATA 17.0 and R programming language.

## Results

**Fig. 1:**
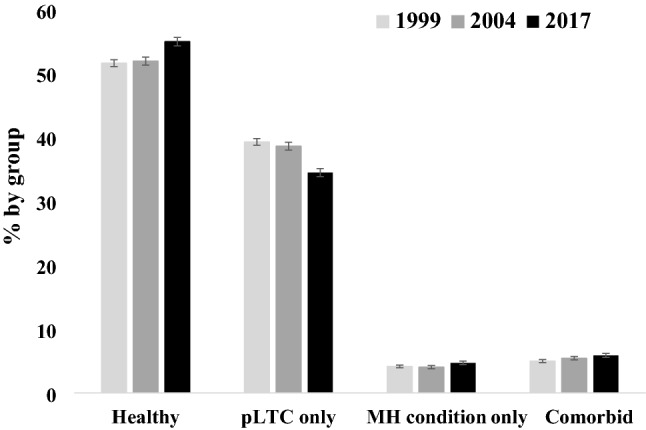
Weighted prevalence estimates of long-term physical health conditions and mental health conditions in children and young people aged 5–15 years in England in 1999 (*N* = 8662), 2004 (*N* = 6401), and 2017 (*N* = 6219) by group. Error bars represent standard errors. MH = mental health; pLTC = long-term physical health condition

Our analysis used data from 8,662 (1999), 6,401 (2004) and 6,219 (2017) CYP from England. The proportion of children with comorbid pLTCs and mental health conditions was 0.050 (95% CI = 0.045, 0.055) in 1999, 0.054 (95% CI = 0.049, 0.060) in 2004, and 0.059 (95% CI = 0.053, 0.065) in 2017. Figure 1 illustrates the weighted prevalence estimates of CYP in the four study groups at the three time points. Prevalence estimates indicated an increase in the proportion of CYP with neither pLTCs or mental health conditions, and a corresponding decrease in the proportion of CYP with pLTCs. Additionally, we showed an increase in the proportion of CYP with mental health conditions and in those with comorbid pLTCs and mental health conditions from 1999 to 2017. Weighted prevalence estimates and 95% CIs for the four study groups are reported in supplementary Table 1.

**Fig. 2:**
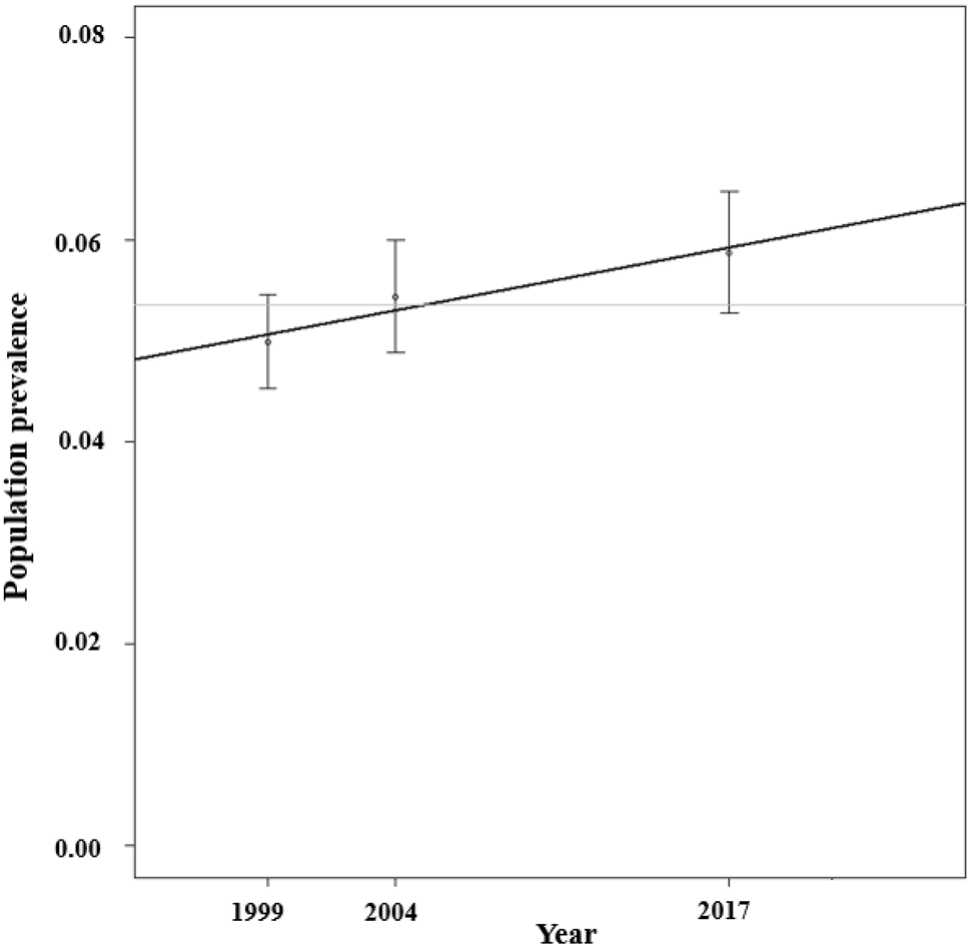
Prevalence estimates of comorbid long-term physical health conditions and mental health conditions in children and young people aged 5–15 years in England from 1999 to 2017

The linear regression model revealed a non-significant effect of time on the proportion of CYP with comorbid pLTCs and mental health conditions from 1999 to 2017 [*B* = 0.0004785; SE = 0.0001256; *p* = 0.163 (Fig. 2)]. Repeating the analyses including the whole Great Britain sample (see supplementary Table 2  for the weighted prevalence estimates) showed a significant increase in the proportion of CYP with comorbid conditions from 1999 to 2017 (*B* = 0.0006; SE = 0.00000312; *p* = 0.003).

## Discussion

To our knowledge, this is the first population-based study to investigate changes in the proportion of CYP with comorbid pLTCs and mental health conditions. Despite the falling prevalence rate of pLTCs, we detected an upward trend (albeit not significant) in comorbid pLTCs and mental health conditions between 1999 and 2017, which is expected given that previous studies suggested a deterioration in the mental health of CYP in the UK during this time [15–17]. We would predict that these rates may well have worsened since 2017, given the effects of Covid-19 pandemic on the mental and physical [18] health of school-aged children [19, 20]. When the analyses were broadened to include CYP from Great Britain, a significant upward trend in the proportion of CYP with comorbid conditions was observed, which may suggest a lower prevalence of comorbid pLTCs and mental health conditions in Scotland and Wales compared to England. We could not directly test this hypothesis as the 2017 survey did not recruit Scottish or Welsh participants. It is also possible that the smaller sample size in England-only analyses reduced the power to detect a change, particularly as so few participants were identified with comorbidities at each time point. Nevertheless, together these findings suggest that CYP with mental health problems have seen no improvements in health over time comparable to the reduction in pLTCs; hence, health disparities are widening. Future studies using larger samples should explore which specific mental health conditions are the most common in CYP with pLTCs, as well as investigate mediators of the presence of comorbid conditions.

Our findings emphasize the need for more effective strategies to identify and support CYP with poor mental health. Combined with the reduction in pLTCs, our study suggests that CYP with physical health needs access support more effectively than CYP with mental health needs; we have yet to obtain ‘parity of esteem’. To halve the number of children with persistent mental health problems, which is the first of the shared goals as stated by the Department of Health and Social Care [21], the identification of problems is required. Even though CYP with poor physical health are in regular contact with primary and secondary health care, which provides opportunities for the identification of symptoms, mental health conditions are often missed [22]. We recently found that CYP with comorbid pLTCs and mental health conditions are more often in contact with primary health care and pediatricians for their mental health than specialist mental health services [Under review]. However, pediatricians only identify a quarter of all cases with a possible psychiatric disorder [22]. Timely delivery of treatment is also critical to prevent mental health conditions from becoming entrenched, yet children waited for a mean of 2 months pre-pandemic for specialist mental health assessment, with many children still not accepted onto waiting lists [23]. This is double the UK government’s 4-week target [24], suggesting that services are overstretched and under-resourced. Improving practitioners’ specific knowledge on childhood mental health conditions as well as introducing routine mental health screening in primary and secondary health care settings could be beneficial [25]. Brief and/or low-intensity (< 6 sessions) psychological interventions based on cognitive behavioral principles [26] have demonstrated the potential to benefit young people with [27] and without [28, 29] a pLTC. These interventions could be delivered at drop-in centers [30], which could be in primary or secondary health care, or at schools [31], to increase access to evidence-based therapy for CYP and their families.

Our analysis benefits from three robust and comparable single-phase, population-based surveys with rigorous mental health assessment using the same measure in each survey. Many previous surveys have used self-/parent report of mental health conditions [32] instead of a multi-informant standardized diagnostic assessment. If a standardized diagnostic assessment is used, surveys often use a two-phase approach with a simple screen initially administered [33], rather than the detailed, one-phase approach in those analyzed here (where each participant had the diagnostic assessment). The survey data we analyzed covered both children and adolescents whereas other similar studies have involved either children only [34] or adolescents only [35]. However, the lengthy gap between 2004 and 2017 assessments may have masked interim time trends. Given the design of the survey, it was not possible to collect objective medical data; thus, pLTCs were parent reported and we lack data on disease severity. Given the likely impact of the COVID-19 pandemic and resulting restrictions, a more recent survey to assess the current level of needs is essential, and we would argue that our estimate is necessarily an underestimate.

In conclusion, the proportion of CYP with comorbid pLTCs and mental health conditions in England remained stable since 1999, highlighting the need to prioritize mental health resources for CYP with physical health comorbidities as they are a particularly vulnerable group.

### Supplementary Information

Below is the link to the electronic supplementary material.

Supplementary file1 (DOCX 21 KB)

## Data Availability

The 1999 and 2004 data are available via application to the UK Data Service (references 4227 and 5269, respectively). The 2017 data are available via NHS Digital’s Data Access Request Service (DARS, reference DARS-NIC-424336-T7K7T-v0.6).
