# Supplementary material for: Trends in comorbid physical and mental health conditions in children from 1999 to 2017 in England
Source: Eur Child Adolesc Psychiatry. 2022 Nov 23;33(1):33–8. doi: 10.1007/s00787-022-02112-5 (PMC9685012; doi:10.1007/s00787-022-02112-5)
Supplement: Supplementary file 1 — Supplementary file1 (DOCX 21 KB) [file 787_2022_2112_MOESM1_ESM.docx]

**Supplementary material**

| Table 1. Weighted prevalence estimates of long-term physical health conditions and mental health conditions in children and young people 5-15 years old in England by group | | | |
| --- | --- | --- | --- |
| Proportion (95 % CI) | | | |
|  | **1999**  N = 8,662 | **2004**  N = 6,401 | **2017**  N = 6,219 |
| Healthy | 0.516 (0.505, 0.526)  *n* = 4,478 | 0.519 (0.507, 0.531)  *n* = 3,314 | 0.550 (0.537, 0.562)  *n* = 3,390* |
| Long-term physical health condition and no psychiatric disorder | 0.393 (0.382, 0.403)  *n* = 3,396 | 0.386 (0.374, 0.398)  *n* = 2,476 | 0.345 (0.332, 0.357)  *n* = 2,160* |
| Psychiatric disorder and no long-term physical health condition | 0.042 (0.037, 0.046)  *n* = 358 | 0.040 (0.036, 0.045)  *n* = 261 | 0.047 (0.042, 0.053)  *n* = 300* |
| Comorbid | 0.050 (0.045, 0.055)  *n* = 430 | 0.054 (0.049, 0.060)  *n* = 350 | 0.059 (0.053, 0.065)  *n* = 375* |
| *Note. CI = confidence interval; N = number; n = number.*  **Please note that Statistical Disclosure Control requirements for the 2017 survey stipulate rules around rounding of numbers which have been followed here.* | | | |

| Table 2. Weighted prevalence estimates of long-term physical health conditions and mental health conditions in children and young people 5-15 years old in Great Britain by group | | |
| --- | --- | --- |
|  | **1999**  N = 10,316 | **2004**  N = 7,261 |
| Healthy | 0.519 (0.510, 0.529)  *n* = 5,382 | 0.524 (0.512, 0.536)  *n* = 3,783 |
| Long-term physical health condition and no psychiatric disorder | 0.390 (0.381, 0.400)  *n* = 4,012 | 0.386 (0.374, 0.397)  *n* = 2,810 |
| Psychiatric disorder and no long-term physical health condition | 0.041 (0.038, 0.045)  *n* = 425 | 0.038 (0.034, 0.043)  *n* = 288 |
| Comorbid | 0.049 (0.045, 0.053)  *n* = 497 | 0.052 (0.047, 0.057)  *n* = 380 |
| *Note. CI = confidence interval; N = number; n = number.* | | |
